# Differential expression of zinc finger CCHC-type superfamily proteins in thyroid carcinoma and their associations with tumor immunity

**DOI:** 10.1186/s13044-023-00185-1

**Published:** 2023-10-17

**Authors:** Yin Yin, Jing Chen, Qian Chen, Hongyan He, Nannan Zhu, Pengcheng Xia, Chunli Yu, Lingyun Meng

**Affiliations:** 1 Department of Clinical Laboratory Medicine, The Second Affiliated Hospital of Shandong First Medical University, Taian, Shandong China; 2 Discipline of Anatomy and Pathology, Shandong First Medical University, Jinan, Shandong China; 3 Department of Clinical Laboratory Medicine, The Affiliated Taian City Central Hospital of Qingdao University, Taian, Shandong China; 4 Department of Clinical Laboratory Medicine, Shandong Provincial Hospital, Shandong First Medical University, Jinan, Shandong China; 5 Department of Pharmacy, Xintai Traditional Chinese Medicine Hospital, Taian, Shandong China

**Keywords:** Thyroid carcinoma, *ZCCHC*, Microenvironment, Immune cell infiltration

## Abstract

**Background:**

The zinc-finger CCHC-type (ZCCHC) superfamily proteins are characterized by the shared sequence CX2-CX4-HX4-C and thought to own high affinity to single-stranded nucleic acids, particularly RNAs. In humans, a total of 24 ZCCHC proteins have been annotated in the HUGO Gene Nomenclature Committee (HGNC, https://www.genenames.org/) database with most of these members involved in multiple steps of RNA metabolism. Many studies have indicated that the *ZCCHC* genes play a regulatory role in the development and progression of solid tumors. To date, the expression pattern and prognostic value of ZCCHC factors in thyroid carcinomas have not been reported.

**Methods:**

Bioinformatics analyses on the functions of ZCCHC factors in thyroid carcinoma (THCA) patients were performed based on various databases, i.e., TCGA, GEPIA, Kaplan-Meier Plotter, and TIMER.

**Results:**

Compared with normal tissues, the expression of *ZCCHC12* mRNA was significantly increased in THCA tissues. And it was associated with the overall survival of THCA patients, based on the Kaplan-Meier Plotter database. Furthermore, the expression levels of all *ZCCHCs* were correlated with tumor stages, implying its high relevance to THCA, specifically its immunity.

**Conclusion:**

The *ZCCHC* genes, represented by *ZCCHC12*, are differentially expressed in THCA staging. These genes are associated with immune infiltration of THCA and identified as the potential therapeutic targets for immunotherapy in THCA patients, which are possible novel biomarkers for the treatment of THCA.

**Supplementary Information:**

The online version contains supplementary material available at 10.1186/s13044-023-00185-1.

## Introduction

The thyroid, the largest endocrine gland in the body, is located in the neck, in an H-shape on both sides of the trachea, resembling a recluse’s armor and more like a pink butterfly with two wings opened. The thyroid gland controls the energy consumption rate and regulates the body’s sensitivity to hormones [1]. Thyroid carcinoma (THCA)is the most common endocrine malignancy, accounting for 1% of all malignancies around, among which papillary carcinoma is the most common type of THCA with a low degree of malignancy and a favorable prognosis [2]. THCA is known to have a higher incidence in women, with a ratio ranging from 1:2 to 1:4 compared to men. Additionally, THCA can develop at any age, although it is more commonly observed in young adults [3]. The known factors causing THCA include high iodine diet [4], radiation [5], and family factors [6, 7]. Although THCA is not very lethal, it still has the potential to recur and metastasize, especially when it is highly malignant, more active management should be given [8]. To treat THCA, oncologists have a variety of strategies, e.g., surgical treatment [9], TSH suppression therapy [10], iodine 131 therapy [11], radiotherapy [12], and molecular targeted therapy [13]. While treatment modalities such as surgery and iodine 131 therapy are now relatively mature, molecular-targeted therapy still requires intensive improvement. Numerous genes that are closely linked to THCA have been identified, including the high expression of vascular endothelial growth factor and its receptor in differentiated THCA [14]. These discoveries have led to the exploration of molecular targets for potential THCA treatments. Additionally, genetic alterations such as the BRAFV600E mutation [15], RET rearrangement [16], and RAS point mutation [17]have been observed in THCA cases. Despite these advancements, immunotherapy for THCA is still considered to be in the clinical research phase [18, 19].

The ZCCHC superfamily proteins are characterized by a common sequence CX2-CX4-HX4-C and are thought to own high affinity with single-stranded nucleic acids, especially the single-stranded RNAs [20]. To date, a total of 24 ZCCHC proteins have been annotated in humans in the HGNC database [21]. It is worth noting that most of the members of this gene family are involved in RNA metabolism and immune function [22]. The *ZCCHC* genes are aberrantly expressed in a variety of solid tumors that take part in the malignant progression of tumors [23, 24]. However, the expression of *ZCCHC* genes in THCA and their roles in disease prognosis and staging have not been studied.

## Materials and methods

### Acquisition of sequencing data and screening of differentially expressed genes (DEGs)

The experimental group contained a total of 512 RNA-seq datasets of thyroid tumors obtained from the Cancer Genome Atlas database (https://portal.gdc.com/). The control group included the RNA-seq data of a total of 59 normal thyroid tissues retrieved from the TCGA databases with the clinical information of these specimens downloaded as well. Screening of DEGs in sequencing data was performed using the Limma package for R software (version: 3.40.2) based on the screening thresholds of adjusted *P* < 0.05 and log_2_ (fold change) > 1 or < − 1.

### Functional enrichment analysis of DEGs

To further investigate the role played by DEGs in tumors, we used the ClusterProfiler package in R to annotate the Gene ontology (GO, http://geneontology.org/) functions of DEGs and to perform the Kyoto Encyclopedia of Genes and Genomes (KEGG, https://www.genome.jp/kegg/) pathway enrichment analysis. GO is a tool commonly used for annotating gene functions, which are categorized into three groups, i.e., molecular functions (MF), biological pathways (BP), and cellular components (CC). KEGG is a database commonly used to annotate metabolic pathways with genes involved.

### Kaplan-Meier plotting of survival curves and the construction of Sankey diagrams

Based on the Kaplan-Meier webserver (http://kmplot.com/analysis/), a total of 512 THCA patients in TCGA were analyzed for overall survival (OS) according to the expression of *ZCCHC* genes with the survival curves plotted based on the statistically significant differences determined by *P* < 0.05. We used the R package maxstat (Maximally selected rank statistics with several *P*-value approximations version: 0.7–25) to calculate the optimal cutoff value for ENSG00000174460 (ZCCHC12). We set the minimum number of samples in one group to be greater than 25% and the maximum number of samples in the other group to be less than 75%. The final optimal cutoff value obtained was 5.3484. Based on this value, we divided the patients into high and low groups.Sankey diagrams were drawn by gg alluvial of the R package, with each column representing a clinical factor (e.g., staging and age) and the connecting lines representing the association of gene expressions with each pair of clinical factors.

### Analysis of immune function by TIMER and correlation with *ZCCHCs*

The TIMER database (http://cistrome.org/TIMER/) [25] was used to perform the analysis of tumor-infiltrating immune cells (TIICs) in TCGA, and the correlation of the immune score in THCA obtained with the abundance of immune infiltration was analyzed based on the expression of *ZCCHC* genes.

### Analysis of Tumor stemness and Tumor mutational burden correlation (TMB)

It has been shown that both the differentiation phenotype of cancer and the characteristics of progenitor/stem cells are associated with cancer progression, and this association score could be calculated by a logistic regression machine learning algorithm (OCLR) [26] used to assess the degree of stemness of a sample by calculating a stemness index based on the transcriptome of the sample. The OCLR algorithm was used to calculate mRNAs, which was an mRNA expression-based feature containing a gene expression profile of 11,774 genes, and we applied Spearman to calculate the correlation and subsequently mapped the stemness index to a range from 0 to 1 using a linear transformation of subtracting the minimum and dividing by the maximum as expressed in the formula (${x}^{*}=\frac{x-min}{max-min}$), where ${x}^{*}$ represented the calculated stemness index, $x$ represented the stemness index before calculation for each specimen, min represented the minimum stemness index, and max represented the maximum stemness index. All these analytical methods were performed using the R software. The distribution of stemness scores and clinical information was plotted, with the top panel representing the distribution of stemness scores from low to high and the bottom panel showing the distribution of ranked clinical features. The correlation analysis between stemness scores and expression of *ZCCHC* genes was performed using Spearman statistics. TMB is often used as an important biomarker for assessing immunotherapy [26], and correlation analysis of TMB and expression of *ZCCHC* genes was performed using Spearman statistics.

### Protein-Protein Interaction Network (PPI) construction

PPI network was constructed based on two databases, i.e., String (https://string-db.org/) and PINA (https://omics.bjcancer.org/pina/). The String database contained data on direct and indirect interactions between proteins. Colored ‘blobs’ (or nodes) indicated proteins with direct interactions, and different colored patterns of connecting lines (or edges) between the ‘blobs’ indicated different types of interactions. The PINA platform was similar to the String database, but with improved analysis of protein interactions according to disease type, especially tumor type, providing greater insight into the function of the proteins in the network, i.e., PINA analysis focuses more on the role that proteins play in tumors.

### Gene location and structure visualization and identification of genes associated with THCA

Both gene location and structure were annotated by TBTOOLS (https://www.tbtools.com/) [26], with the protein files and human reference genome files downloaded from the NCBI (https://www.ncbi.nlm.nih.gov/) database. The word “motif” described a recurring pattern, and sequence motifs often showed a recurring pattern in DNA and were assumed to have a biological function. Furthermore, it was often the binding site of a protein that shows sequence specificity (e.g., transcription factors) or is involved in an important biological process, and MOTIF analysis was performed based on the TBTOOLS database to provide comprehensive, user-friendly information on all annotated and predicted human genes. The GENECARDS database (https://www.genecards.org/) was used to search for genes associated with THCA, and the top 25 genes with the highest correlation coefficients were defined as genes closely related to THCA, and were eventually used in the correlation analysis with the *ZCCHC* gene family.

### Forest plot of survival factors and expression and staging analysis

Univariate cox regression analyses were performed with forest plots used to display each variable (i.e., p-value, HR, and 95% CI) via the “forest plot” package in R software. The distribution of expression of 12 genes in tumor and normal tissues was determined by Kruskal-Wallis test for significance in multiple sample groups.

### Cell culture

The human normal thyroid epithelial cell line NTHYORI 3–1, and human papillary thyroid cancer cell line TPC1 were provided by the American Type Culture Collection (ATCC; Manassas, VA, USA). NTHY-ORI 3–1 and TPC-1 cells were cultured in RPMI-1640 containing 10% fetal bovine serum (FBS, Gibco, Carlsbad, CA); SW579 cells were cultured in Leibovitz’s L-15 medium containing 10% FBS; 8505 C cells were cultured in DMEM containing 10% FBS. All cells were cultured in a 5% ­CO2 incubator at 37 °C.

### Validation of *ZCCHCs* genes

The quantitative real-time PCR (qRT-PCR) analysis was performed to verify the expression patterns of *ZCCHC* genes revealed in the microarray analysis. The primer sequences were synthesized by Tsingke Biotechnology Co., Ltd., Qingdao, China (Table S1). Trizol (Thermo Fisher Scientific Inc., MA, USA) method was employed to extract the total RNA from cells in each group according to the manufacturer’s protocol. The total RNA (1 µg) was reverse-transcribed to cDNA by use of PrimeScript RT Reagent Kit with gDNA Eraser (Accurate Biotechnology Co., Ltd., Hunan, China). All genes involved in the experiment were examined by a quantitative real-time PCR amplifier (Applied Biosystems QuantStudio 5, ABI Company, Oyster Bay, New York, USA) with SYBR® Premix Ex Taq (Accurate Biotechnology Co., Ltd., Hunan, China). PCR procedure: pre-denaturation at 95 °C for 30 s, 40 cycles of denaturation at 95 °C for 5 s, annealing at 60 °C for 30 s, extension at 72 °C for 30 s, and finally melting at 95 °C for 30 s.

### Validation of ZCCHC gene family protein expression by immunohistochemical staining

We searched The Human Protein Atlas database (https://www.proteinatlas.org/) for immunohistochemical data from THCA patients to confirm the differential expression of proteins encoded by the *ZCCHC* gene family, not all proteins encoded by the *ZCCHC* gene family were included in the database and we present the results retrieved.

### Statistical methods

The statistical tool R version:3.40.2 was used for all statistical analyses. Sequencing data of TCGA were tested for normality and homogeneity of variance before analysis. Comparisons between two groups were made using the t-test, and correlations between two groups were determined using the Spearman test. A *P*-value less than 0.05 was considered statistically significant.

## Results

### Identification and functional enrichment analysis of DEGs involved in THCA

**Fig. 1:**
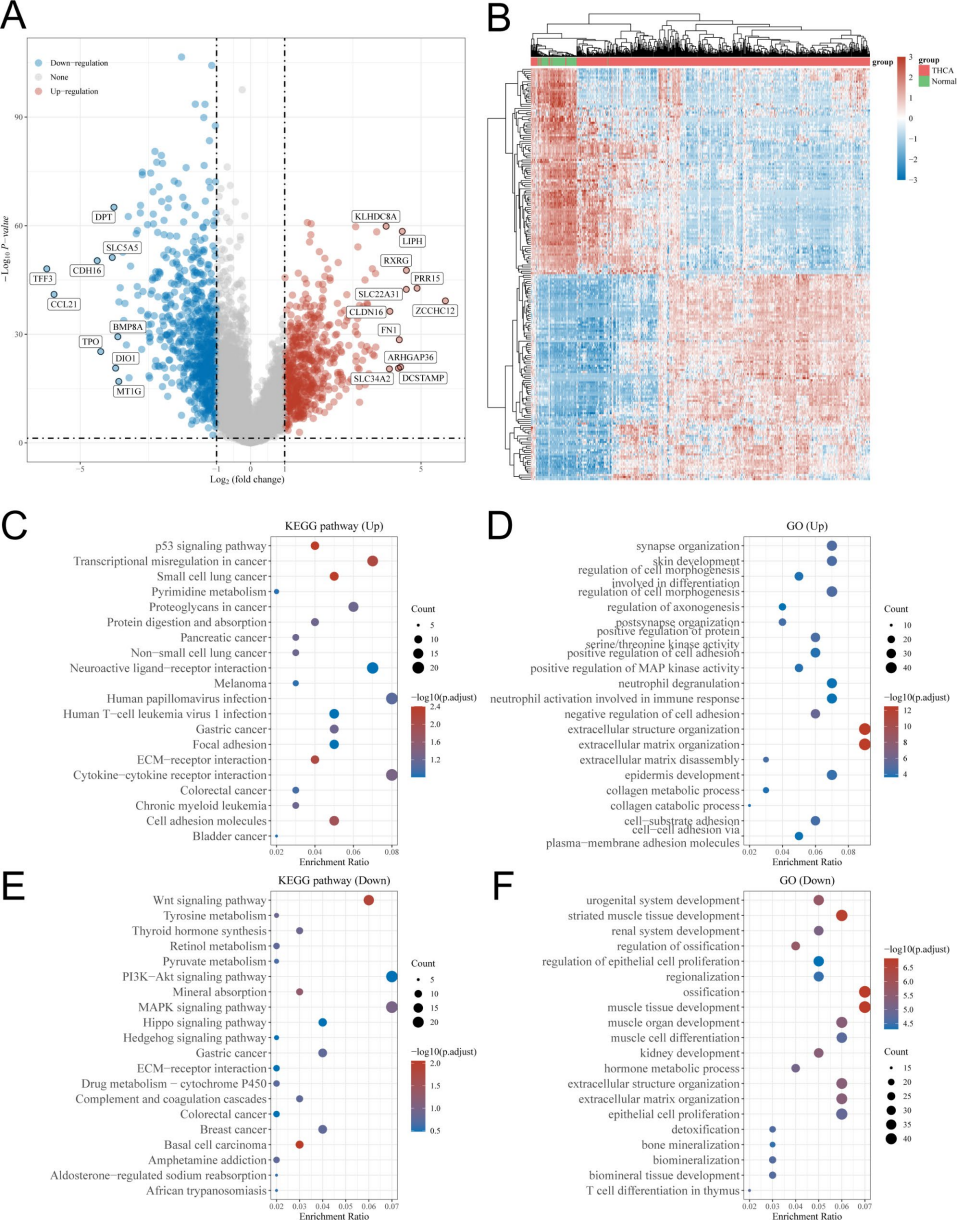
Screening for differentially expressed genes (DEGs) in THCA and functional enrichment analysis of these DEGs. **A** Volcano map for DEG screening with volcanoes plotted using fold change and corrected p-values. Red dots in the schematic indicate genes that are significantly differentially up-regulated, blue dots indicate genes that are significantly differentially down-regulated, and grey dots indicate genes that are not significantly regulated. **B** Heat map for clustering analysis of DEGs with different colors representing the expression trends in different tissues. Due to the large number of DEGs, only 50 up-regulated and 50 down-regulated genes with the greatest differential change are shown here. **C** KEGG analysis of highly expressed DEGs. **D** GO analysis of highly expressed DEGs. **E** KEGG analysis of DEGs with low expression. **F** GO analysis of DEGs with low expression. The different colors represent the significance of the differential enrichment results with the larger values representing smaller FDR values. The size of the circles represents the number of enriched genes with the larger circles representing the larger number of genes

The “Limma” package was used to analyze the DEGs between THCA and normal tissues. A total of 557 up-regulated and 746 down-regulated DEGs were obtained, with *ZCCHC12* identified as the most highly expressed gene (Table S2). These results were presented in the volcano and heat maps (Fig. 1A, B). And then, we conducted function analysis based on up-and down-regulated genes respectively. GO functions annotated by the up-regulated genes included synapse organization, skin development, regulation of cell morphogenesis involved in differentiation, regulation of cell morphogenesis, regulation of axonogenesis, etc. (Fig. 1D). While top 20 KEGG metabolic pathways enriched by these genes included p53 signaling pathway, Transcriptional misregulation in cancer, Small cell lung cancer, Pyrimidine metabolism, Proteoglycans in cancer, etc. (Fig. 1C). GO terms annotated by down-regulated genes were urogenital system development, striated muscle tissue development, renal system development, regulation of ossification, regulation of epithelial cell proliferation, etc. (Fig. 1F). And the top 20 KEGG metabolic pathways enriched by down-regulated genes included Wnt signaling pathway, Tyrosine metabolism, Thyroid hormone synthesis, Retinol metabolism, Pyruvate metabolism, etc. (Fig. 1E).

### Expression of *ZCCHC12* and its correlation with THCA survival and staging

**Fig. 2:**
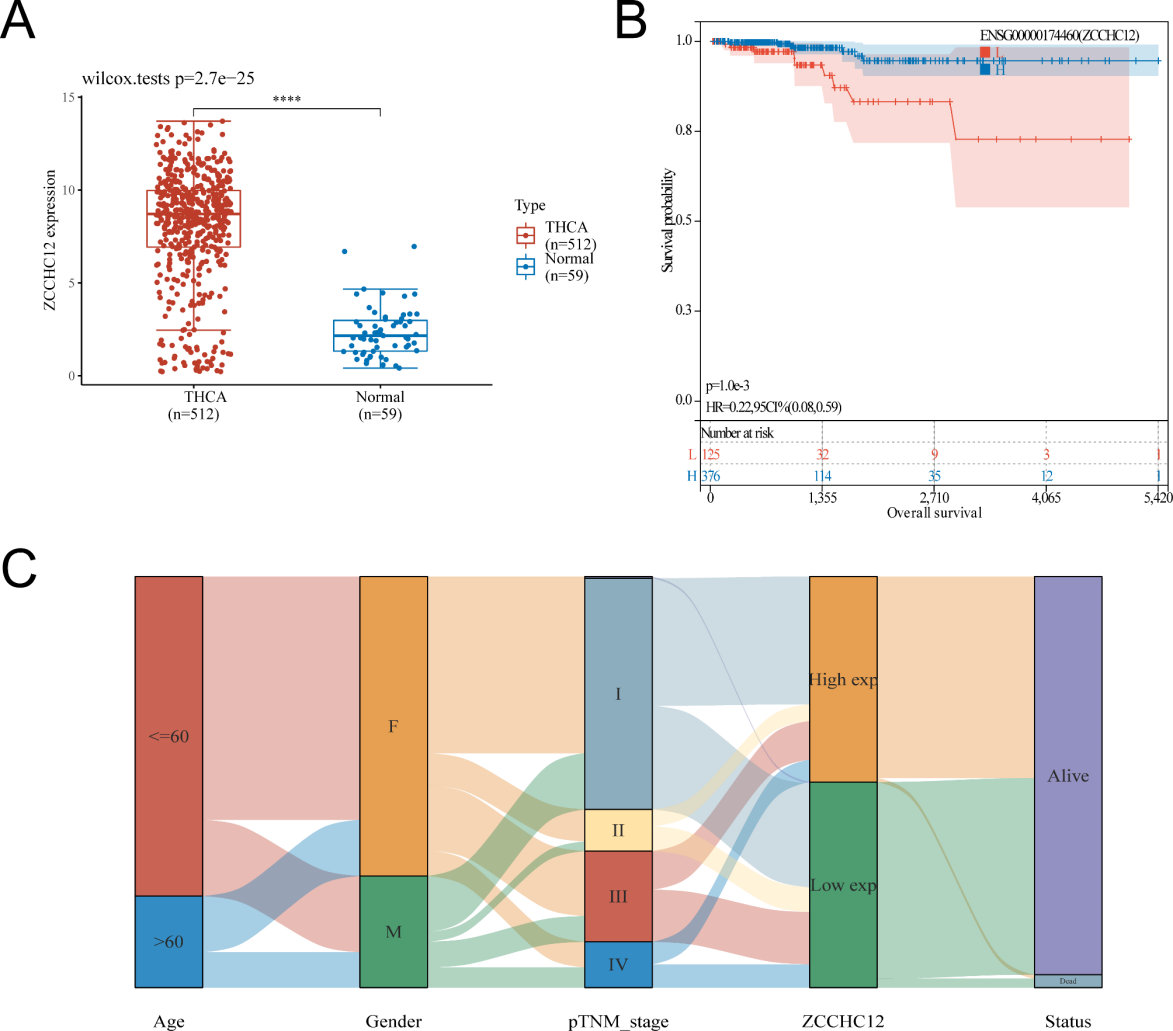
*ZCCHC12* expression in THCA and its correlation with survival and staging. **A***ZCCHC12* expression in THCA. **B** K-M analysis of *ZCCHC12* and survival in THCA. **C** Sankey diagram of age, sex, stage, *ZCCHC12* expression, and survival status in THCA. Symbols “****” indicate the significant difference based on *P* < 0.0001

As Fig. 2A shows, the expression of *ZCCHC12* was significantly higher in THCA than in normal controls. And then, we collected 501 THCA patients and categorized them into the high (376) and low (125) groups based on *ZCCHC12* expression levels. The Kaplan-Meier curves revealed that those patients with high *ZCCHC12* expression showed a higher survival prognosis (*P* = 1.0E-3; Fig. 2B). And the Sankey diagram (Fig. 2C) showed that the majority of THCA patients were younger than 60 years old, with more female patients than male patients. Patients at tumor stage I accounted for the majority of patients, whereas the smallest proportion of patients was identified in stage II. Notably, most patients with high *ZCCHC12* expression are at stage I, while low *ZCCHC12* expression patients are at stages II and III. Correspondingly, a higher proportion of patients with low *ZCCHC12* expression were among the patients who died (Table S3). What needs to be clarified here is that the expression of *ZCCHC12* is higher in THCA patients compared to normal individuals. However, after being diagnosed with THCA, the high expression of *ZCCHC12* is actually beneficial for the survival of patients.

### Correlation of *ZCCHC12* with Tumor immunity, Tumor stemness, and TMB, and the PPI network with the ZCCHC gene family

**Fig. 3:**
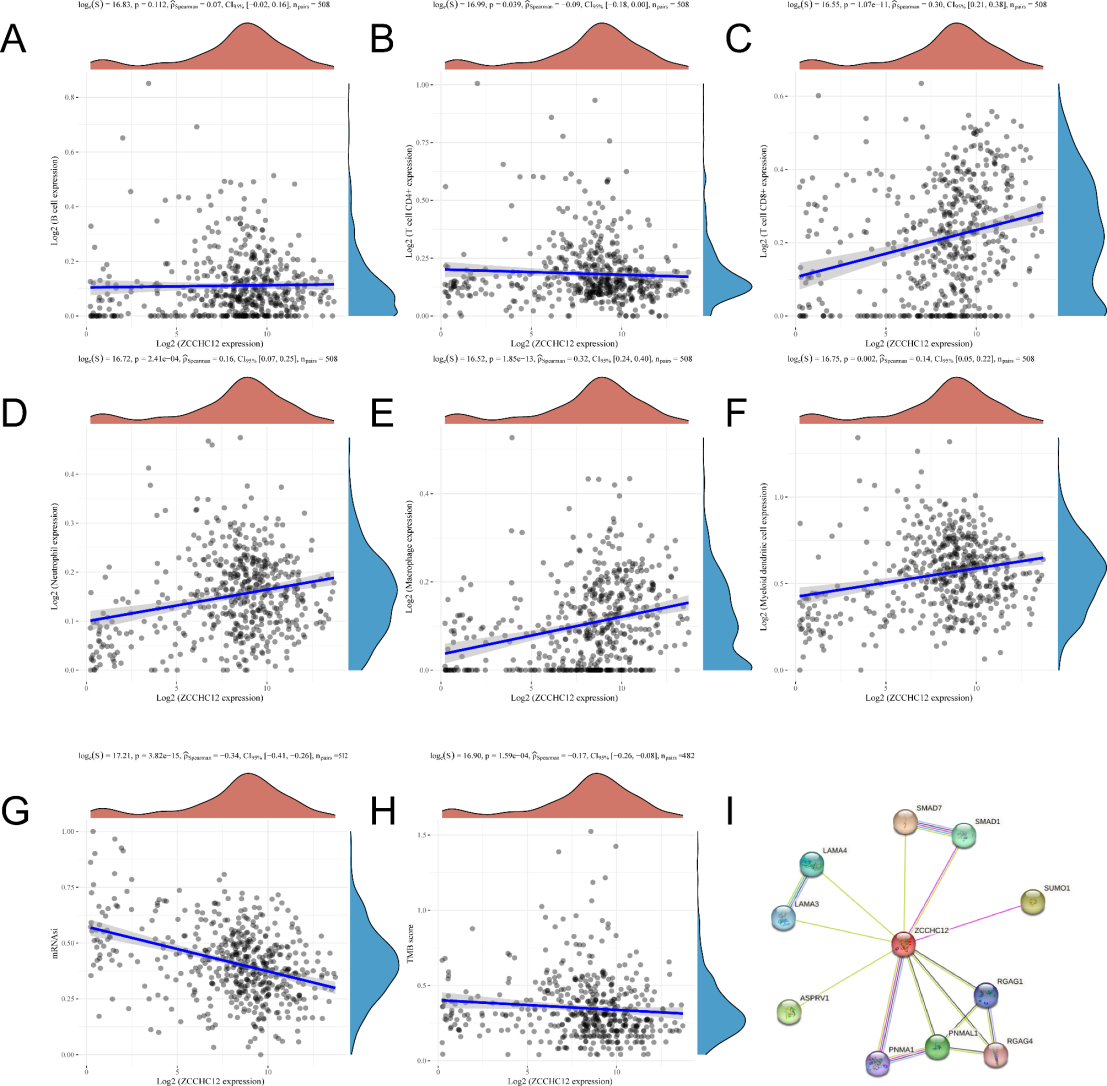
Correlation analysis of *ZCCHC12* with tumor immunity, tumor stemness, and TMB, and the PPI network for the *ZCCHC* gene family. **A-F** Correlation of *ZCCHC12* with different types of immune cells in THCA. **G** Correlation of *ZCCHC12* with tumor stemness score in THCA. **H** Correlation of *ZCCHC12* with tumor mutational load in THCA. **I** PPI network of proteins interacting with *ZCCHC12*. Correlation analyses are performed using Spearman statistics, with the horizontal coordinates representing gene expression and the vertical coordinates representing the immune score distribution. The density curve (right) represents the trend in score distribution, the upper density curve is the trend in gene distribution, and the uppermost value represents the *P*-value of the correlation analysis

In THCA, the *ZCCHC12* expression was not correlated with B-cell expression (*P* = 0.112; Fig. 3A) but was negatively correlated with CD4 + T cells (*P* = 0.039; correlation coefficient = − 0.09; Fig. 3B) and positively correlated with CD8 + T cells (*P* = 1.07e-11; correlation coefficient = 0.30; Fig. 3C), neutrophils (*P* = 2.41e-04; correlation coefficient = 0.16; Fig. 3D), macrophages (*P* = 1.85e-13; correlation coefficient = 0.32; Fig. 3E), and dendritic cells (*P* = 0.02; correlation coefficient = 0.14; Fig. 3F).And the *ZCCHC12* expression was also negatively correlated with both tumor stemness (*P* = 3.82e-15; correlation coefficient = − 0.34; Fig. 3G) and tumor mutational load (*P* = 1.59e-04; correlation coefficient = − 0.17; Fig. 3H). The PPI network analysis revealed a total of 10 proteins associated with ZCCHC12, including ASPRV1, LAMA3, LAMA4, PNMA1, PNMAL1, RGAG1, RGAG4, SMAD1, SMAD7, and SUMO1 (Fig. 3I, Table S4).

### Chromosomal location and MOTIF structure of the *ZCCHC* gene family and its expression and correlation with THCA

**Fig. 4:**
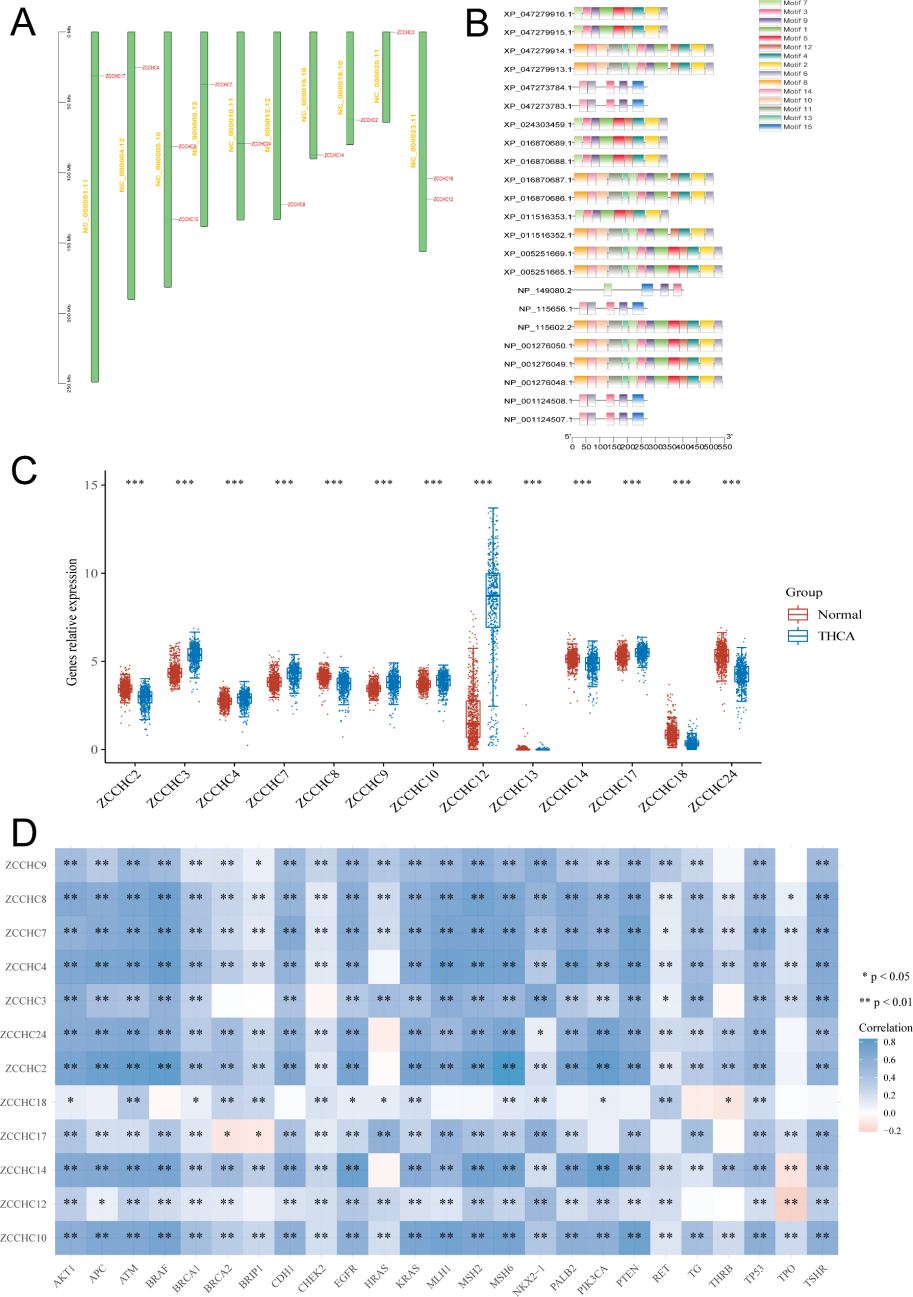
Chromosomal location and MOTIF structure of the *ZCCHC* gene family and their expressions and correlations with THCA. **A** Location of the *ZCCHC* gene family on the chromosomes. **B** Motif structure of the *ZCCHC* gene family. **C** Expression of the *ZCCHC* gene family in THCA. **D** Correlation of the *ZCCHC* gene family with THCA-related genes. *, *P* < 0.05; **, *P* < 0.01; ***, *P* < 0.005

Except for *ZCCHC* genes that were either not expressed in THCA or unstably expressed, a total of 12 genes were retained, including *ZCCHC17* on chromosome 1, *ZCCHC4* on chromosome 4, both *ZCCHC9* and *ZCCHC10* on chromosome 5, *ZCCHC7* on chromosome 9, *ZCCHC24* on chromosome 10, *ZCCHC8* on chromosome 12, *ZCCHC14* on chromosome 16, *ZCCHC2* on chromosome 18, *ZCCHC3* on chromosome 20, and both *ZCCHC12* and *ZCCHC18* on chromosome X (Fig. 4A). Motif analysis showed that these 12 genes consisted of 15 progenitors and belonged to the same gene family (Fig. 4B). The expressions of *ZCCHC* genes in THCA were all statistically different, with the most significant difference detected in *ZCCHC12* (Fig. 4C). The top 25 genes associated with THCA were identified based on the GENECARDS database with the correlation between the *ZCCHC* gene family and these top 25 genes analyzed, showing that most of these genes were related to each other (Fig. 4D, Table S5).

### Association of the *ZCCHC* gene family with THCA survival and staging

**Fig. 5:**
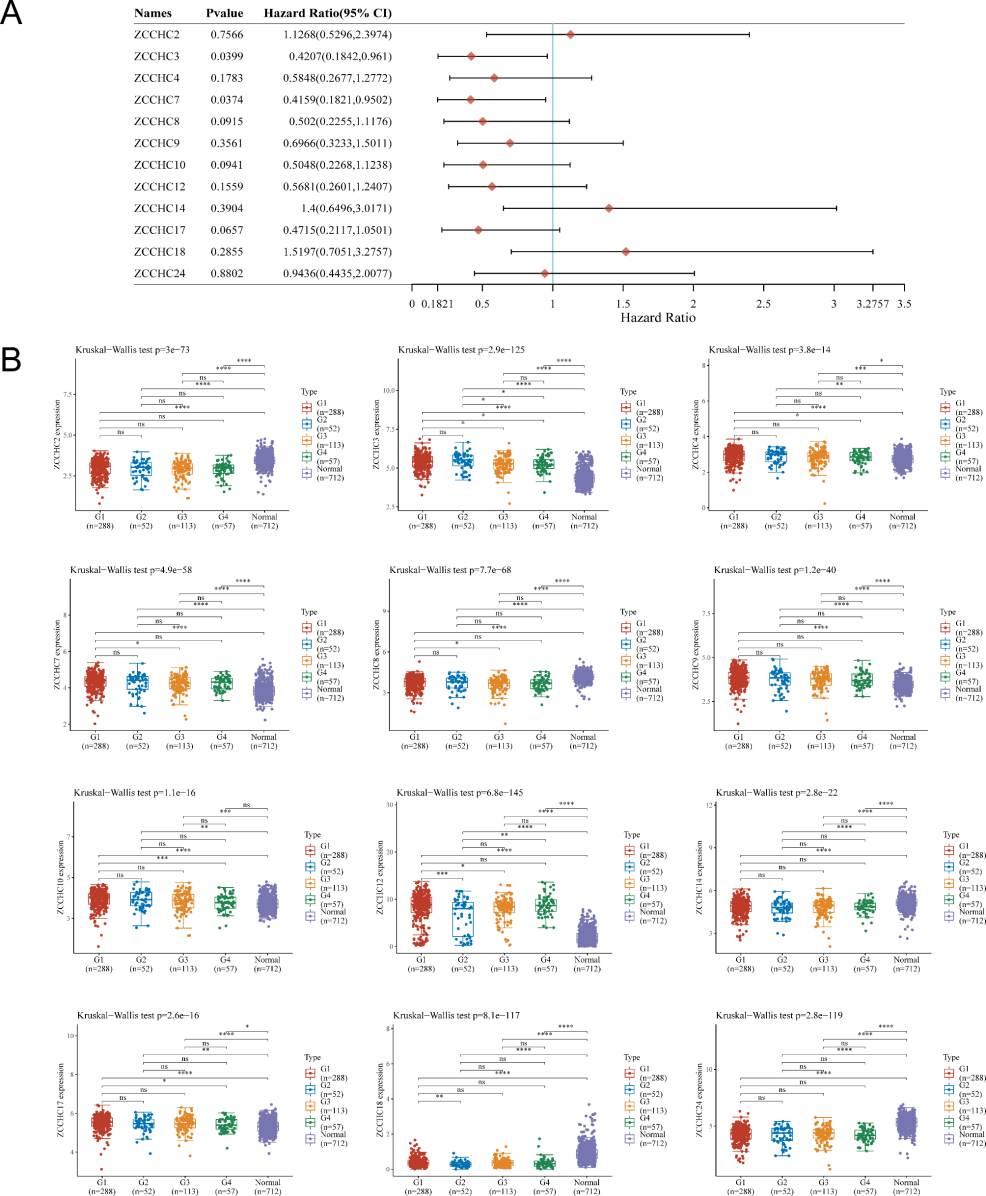
Association of the *ZCCHC* gene family with THCA survival and staging. **A** Plot of one-way cox analysis of the *ZCCHC* gene family and survival in THCA. **B** Differential expression of the *ZCCHC* gene family at different stages in THCA. The horizontal coordinates represent the different groups of samples (G1 = stage I), the vertical coordinates represent the distribution of the gene expression, the different colors represent different groups, and the numbers in the upper left corner represent the significant *p*-values. *, *P* < 0.05; **, *P* < 0.01; ***, *P* < 0.005; ****, *P* < 0.001

Analysis of the line graphs revealed that only *ZCCHC3* and *ZCCHC7* showed an effect on survival based on the univariate COX, but no effect on their association with the other 10 genes (Fig. 5A). Analysis of the expression of the *ZCCHC* gene family in different THCA stages revealed that all *ZCCHC* genes differed at various stages (Fig. 5B). *ZCCHC3* shows a gradual decline in expression with the progression of THCA. However, it is similar to *ZCCHC12* and exhibits higher expression levels in the THCA group compared to the control group, showed strong potential as a biomarker of disease progression (Table S6).

### Diagnostic efficiency of the *ZCCHC* gene family for THCA

**Fig. 6:**
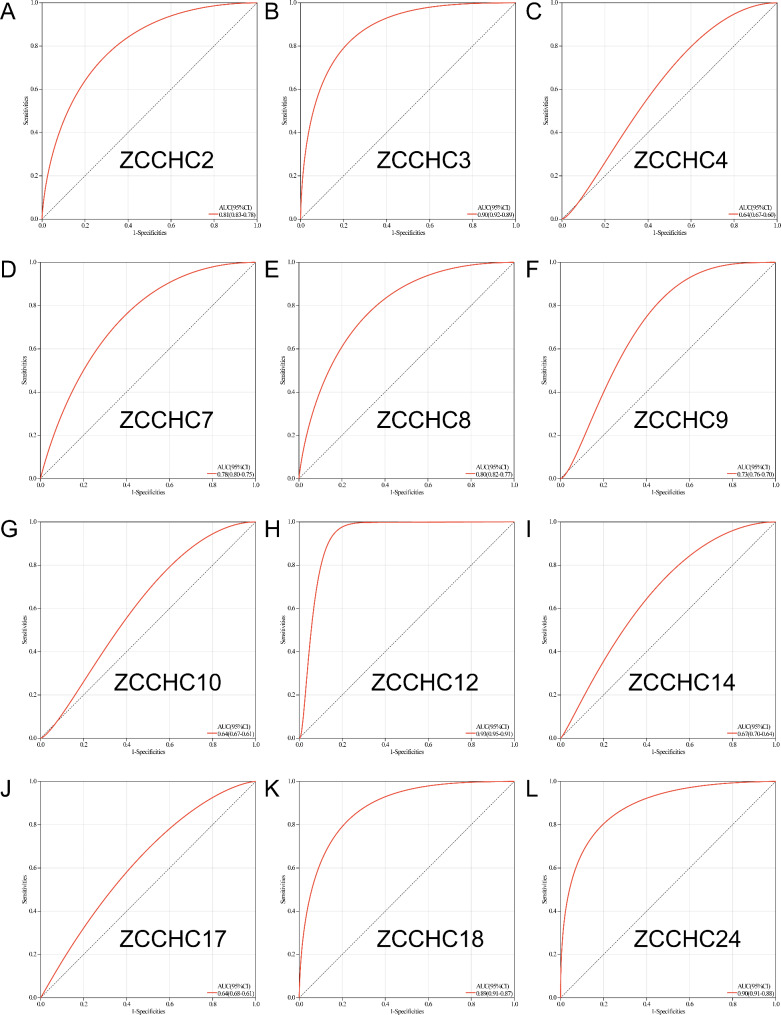
ROC curves of the *ZCCHC* gene family members for THCA, including **A***ZCCHC2*, **B***ZCCHC3*, **C***ZCCHC4*, **D***ZCCHC7*, **E***ZCCHC8*, **F***ZCCHC9*, **G***ZCCHC10*, **H***ZCCHC12*, **I***ZCCHC14*, **J***ZCCHC17*, **K***ZCCHC18*, and **L***ZCCHC24*.

To evaluate the diagnostic efficiency of the *ZCCHC* gene family for THCA, the receiver operating characteristic (ROC) curves were constructed (Table S7), showing that the AUC value of *ZCCHC2* was 0.81 (Fig. 6A), 0.90 for *ZCCHC3* (Fig. 6B), 0.64 for *ZCCHC4* (Fig. 6C), 0.78 for *ZCCHC7* (Fig. 6D), 0.80 for *ZCCHC8* (Fig. 6E), 0.73 for *ZCCHC9* (Fig. 6F), 0.64 for *ZCCHC10* (Fig. 6G), 0.93 for *ZCCHC12* (Fig. 6H), 0.63 for *ZCCHC14* (Fig. 6I), 0.64 for *ZCCHC17* (Fig. 6J), 0.89 for *ZCCHC18* (Fig. 6K), and 0.90 for *ZCCHC24* (Fig. 6L).

### Correlation of the *ZCCHC* gene family with immunity and variations of Tumor stemness in THCA

**Fig. 7:**
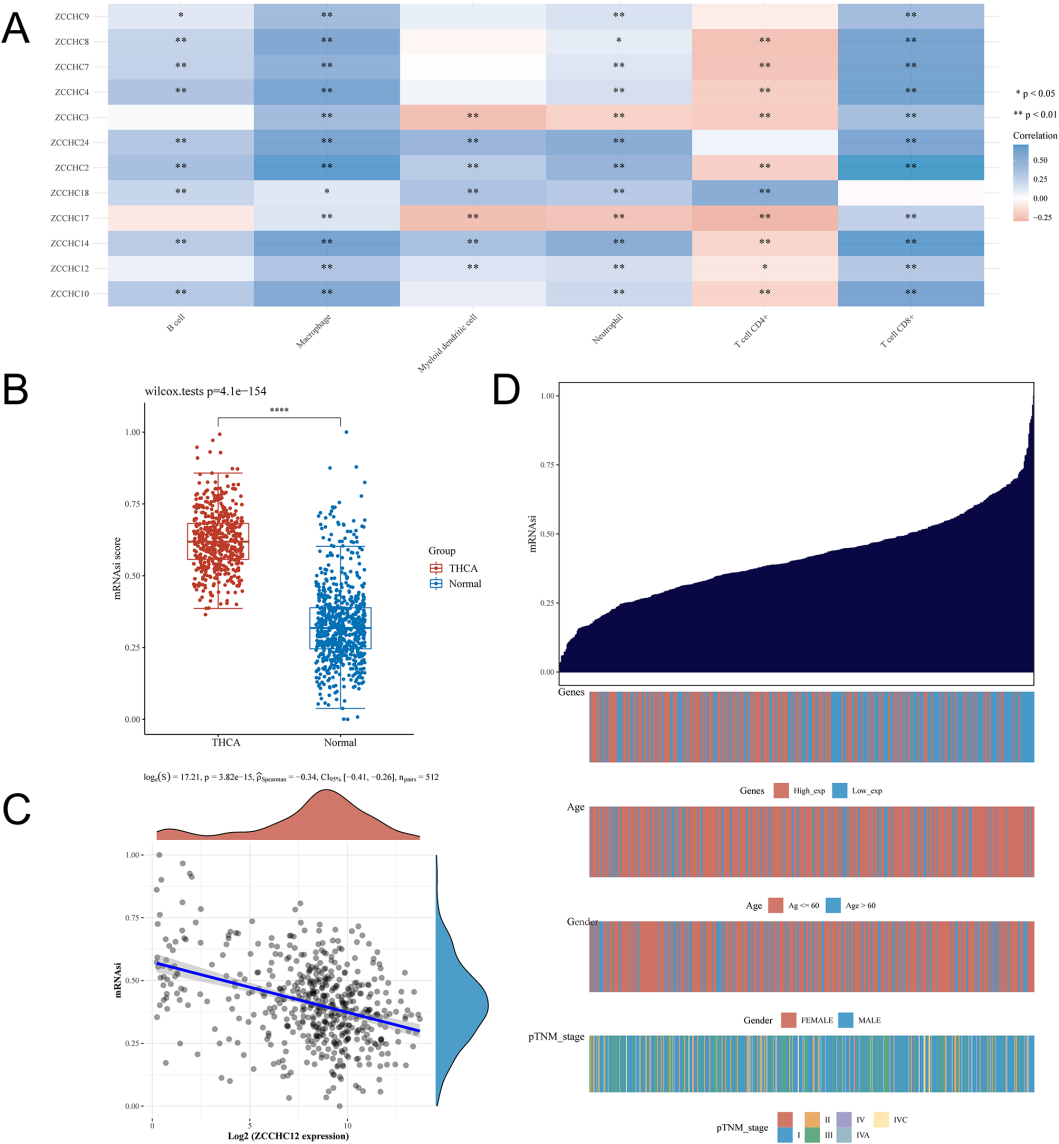
Correlation of the *ZCCHC* gene family with immune cells in THCA and the correlation of tumor stemness differences in THCA with *ZCCHC12*. **A** Correlation of the *ZCCHC* gene family with immune cells. **B** Tumor stemness variations in THCA. **C** Tumor stemness variations in THCA and their correlations with *ZCCHC12*. **D** Tumor stemness distribution in THCA in relation to age, sex, stage, and *ZCCHC12*. *, *P* < 0.05; **, *P* < 0.01; ***, *P* < 0.005; **** *P* < 0.001

In THCA, the immune scores were assessed based on the TIMER database (Table S8), and the correlations between the *ZCCHC* gene family and immune cells were investigated (Fig. 7A). The results revealed that *ZCCHC9* showed positive correlations with B cells, macrophages, neutrophils, and CD8 + T cells; *ZCCHC8* was positively correlated with B cells, macrophages, neutrophils, and CD8 + T cells, and negatively correlated with CD4 + T cells; *ZCCHC7* was positively correlated with B cells, macrophages, neutrophils, and CD8 + T cells, and negatively correlated with CD4 + T cells; *ZCCHC4* was positively correlated with B cells, macrophages, neutrophils, and CD8 + T cells, and negatively correlated with CD4 + T cells; *ZCCHC3* was positively correlated with macrophages and CD8 + T cells, and negatively correlated with dendritic cells, neutrophils, and CD4 + T cells; *ZCCHC24* showed positive correlation with B cells, macrophages, dendritic cells, neutrophils, and CD8 + T cells; *ZCCHC2* showed positive correlation with B cells, macrophages, dendritic cells, neutrophils, CD8 + T cells, and CD4 + T cells; *ZCCHC18* was positively correlated with B cells, macrophages, dendritic cells, neutrophils, and CD4 + T cells; *ZCCHC17* was positively correlated with macrophages and CD8 + T cells and negatively correlated with dendritic cells, neutrophils, and CD4 + T cells; *ZCCHC14* was positively correlated with B cells, macrophages, dendritic cells, neutrophils, and CD8 + T cells and negatively correlated with CD4 + T cells; *ZCCHC12* was positively correlated with macrophages, dendritic cells, neutrophils, and CD8 + T cells and negatively correlated with CD4 + T cells; *ZCCHC10* was positively correlated with B cells, macrophages, neutrophils, and CD8 + T cells and negatively correlated with CD4 + T cells. The tumor stemness was significantly higher in THCA compared to normal controls (*P* = 4.1e-154; Fig. 7B). *ZCCHC12* was negatively correlated with THCA tumor stemness (*P* = 3.82e-15; correlation coefficient = − 0.34; Fig. 7C). A pattern plot of tumor stemness with *ZCCHC12* expression, age, gender, and the stage was constructed by collating THCA clinical data and tumor stemness scores in TCGA. The results revealed lower *ZCCHC12* expressions when the tumor stemness was elevated, more people aged > 60 years old, no significant difference in gender distribution, and a high number of patients at stages higher than I (Fig. 7D).

### GO analysis and PPI network of the *ZCCHC* gene family

**Fig. 8:**
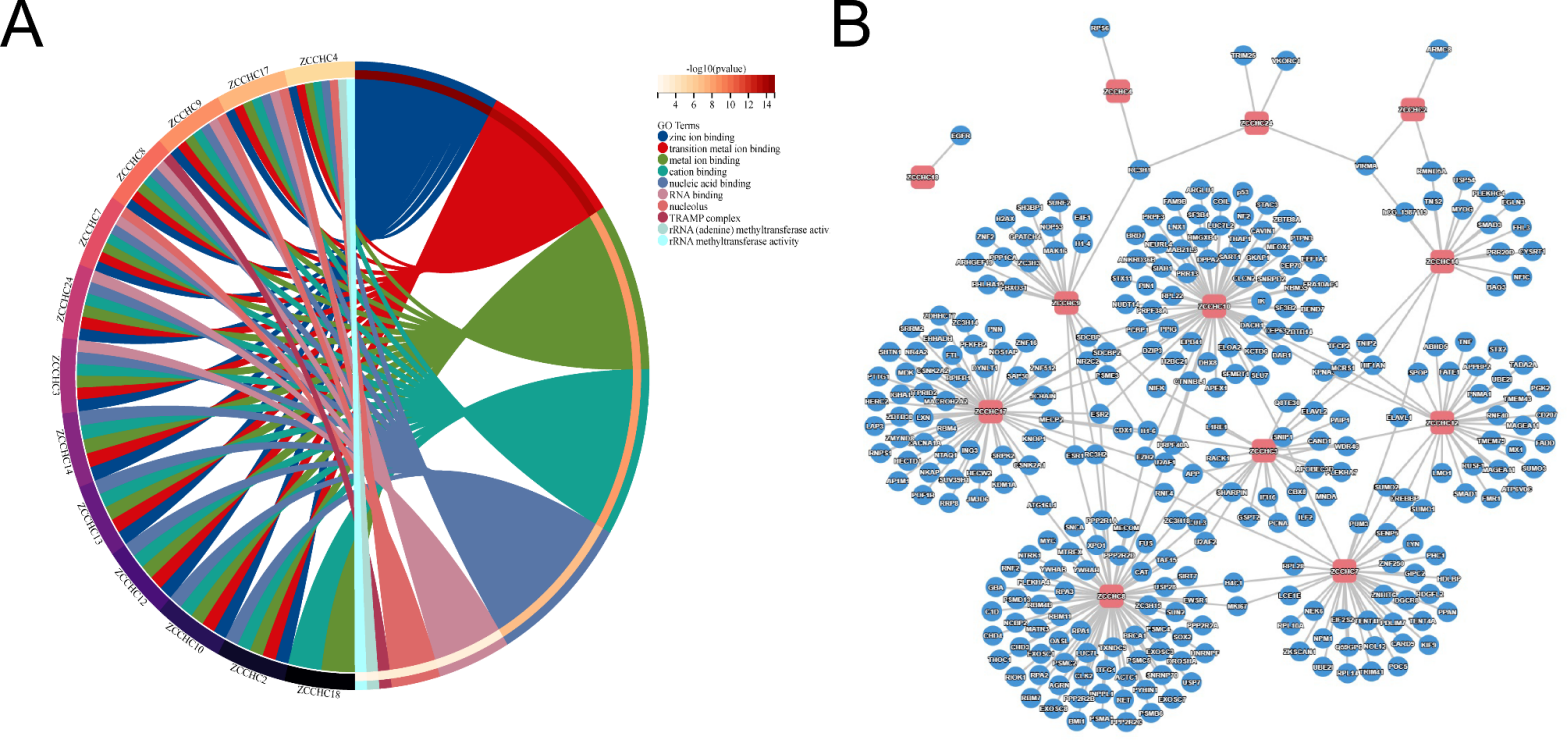
GO analysis **(A)** and PPI network **(B)** of the *ZCCHC* gene family

To analyze the function of the *ZCCHC* gene family (Table S9), the GO annotation analysis was performed based on the *ZCCHC* gene family, showing the differences in the zinc ion binding, transition metal ion binding, metal ion binding, cation binding, nucleic acid binding, RNA binding, Nucleolus, TRAMP complex, rRNA (adenine) methyltransferase activity, and rRNA methyltransferase activity pathways (Fig. 8A). The results showed that most of the *ZCCHC* gene family members except for *ZCCHC18* were interlinked (Fig. 8B).

### Validation of ZCCHC gene family genes and proteins expression

**Fig. 9:**
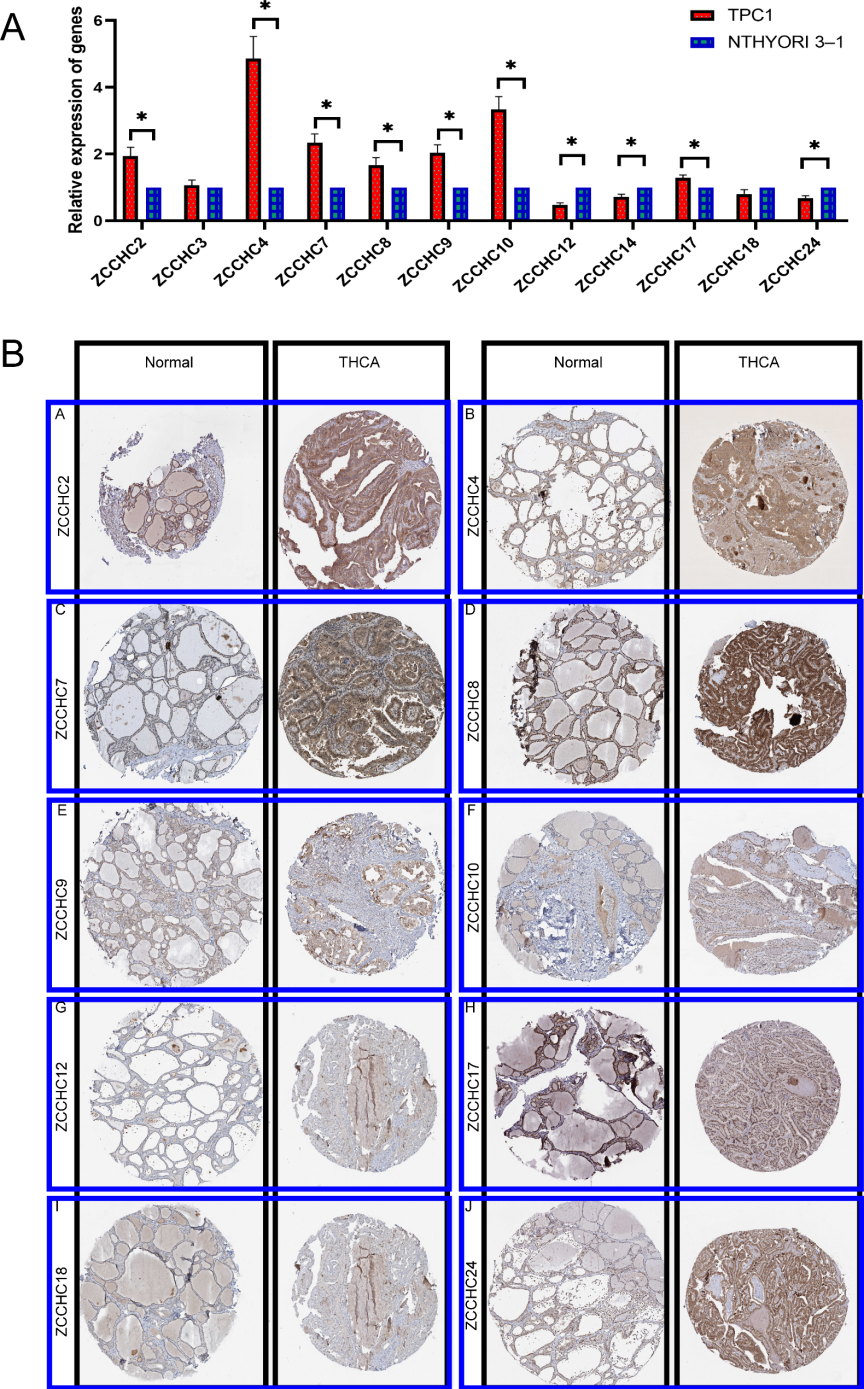
Validation of ZCCHC gene family genes and proteins expression. **A** ZCCHC gene family genes are differentially expressed in TPC1 and NTHYORI 3 − 1 cells. **B** Differential expression of immunohistochemistry of the ZCCHC gene family in The Human Protein Atlas database

We also verified the differential expression of ZCCHC gene family genes in commonly used cell lines for thyroid cancer, and all genes were differentially expressed except *ZCCHC3* and *ZCCHC18* (Fig. 9A). The results of immunohistochemistry confirmed that ZCCHC2, ZCCHC4, ZCCHC7, ZCCHC8, ZCCHC9, ZCCHC10, ZCCHC12, ZCCHC17, ZCCHC24 were higher in THCA patients than in normal controls; ZCCHC18 was lower in THCA patients than in normal controls (Table S10, Fig. 9B). Before experimental validation, we obtained the ZCCHCs gene expression matrix of THCA cell lines from the CCLE database (https://portals.broadinstitute.org/ccle/about). Through analysis, we found that differences in gene expression between cells and gene expression in patients are commonly observed. TPC-1 and Nthy-ori-3 cells are widely used cell lines for THCA research, and we believe it is reasonable to use them to validate the differential expression of the ZCCHC gene family.

## Discussion

Many studies have indicated that the ZCCHC genes play a regulatory role in the development and progression of solid tumors, but the expression pattern and prognostic value of ZCCHC factors in thyroid carcinomas have not been reported at present. In this study, we analyzed data derived mainly from the TCGA database. Considering the number of THCAs for some specific pathological types is very small, we examined the THCA category as a whole, without differentiating the data according to the pathological type of THCA, to investigate the role played by the *ZCCHC* gene family [27].

The *ZCCHC* gene family is now known as being involved in the metabolic mechanisms of messenger RNA (mRNA), microRNA (miRNA), and ribosomal RNA (rRNA) in humans, playing an important role in tumors. For instance, *ZCCHC4* disrupts DNA damage-induced apoptosis to promote resistance to cancer chemotherapy [28], *ZCCHC9* regulates the JNK pathway to enhance proliferation and invasion in lung cancer [24], *ZCCHC12*, on the other hand, regulates the PI3K/AKT pathway to promote osteosarcoma progression [29], *ZCCHC13* induces hepatocellular carcinoma through the regulation of DNA methylation and AKT/ERK signaling pathways [30], In non-small cell lung cancer, *ZCCHC14* controls proliferation and invasion via the MAPK-P38 signaling pathway [31], Finally, *ZCCHC17* serves as a biomarker for predicting prognosis and immunotherapy effectiveness in hepatocellular carcinoma [32]. However, studies on the relevance of the *ZCCHC* family with tumor immunity are still lacking. Therefore, we investigated the functions and associations of the *ZCCHC* gene family with tumor immunity of THCA. The immune cell score, the tumor cell “stem cell index,“ and TMB are three important indicators used in studying and assessing the effectiveness of immunotherapy in THCA. The immune cell score generally refers to the proportion of immune cells in the tumor tissue, which is often surrounded by a large number of immune cells in and around the tumor, and these immune cells are inextricably linked to each other as well as to the tumor cells and immune cells. The tumor cell ‘stem cell index’ concept holds promise for the future development of novel therapies to inhibit cancer progression [33]. The up-regulation of tumor cell stemness directs tumor cells towards tumor stem cells, while the down-regulation of tumor cell stemness indicates that tumor cells are forced to differentiate or even to apoptose. TMB can indirectly reflect the ability and extent of tumor production of neoantigens and predict the efficacy of immunotherapy for a wide range of tumors [34]. Therefore, we performed the immune correlation studies to focus on these three indicators.

We first screened the TCGA database for DEGs in THCA sequencing data to identify *ZCCHC12* as the most differentially expressed gene involved in THCA. Studies have shown that *ZCCHC12* down-regulation significantly inhibited the colony formation, migration, and invasion, in an in vitro assay in papillary thyroid cancer cells [35]. These results were consistent with those revealed in our study, validating the experimental approach used in our study to perform the differential gene analysis to uncover clinically significant DEGs. The DEGs obtained were then analyzed for functional annotation and enrichment analysis of metabolic pathways, showing that the cell adhesion molecules were found to be linked to THCA. These results were consistent with those previously reported, showing that members of cell adhesion molecules have been detected in various cancers and shown to be involved in cancer growth and invasion [36].

We then showed that *ZCCHC12* was correlated with survival and staging of THCA and correlated with both immune score and tumor stemness as well as mutational load in THCA, with many genes associated with immune responses in THCA. For example, studies showed that high expression of *SIGLEC15* mRNA was positively associated with many immune pathways and its upregulation was positively associated with enhanced immune scores, stromal scores, and estimated scores [37]. These results were consistent with the expression pattern of *ZCCHC12* revealed in our study. Furthermore, studies have shown that *ZCCHC12* promotes the progression of osteosarcoma via PI3K/AKT pathway [29]. In our study, the PPI network revealed 10 proteins that interacted with *ZCCHC12* with many of these proteins closely associated with THCA. These findings revealed in our study were consistent with those previously reported. For example, studies have shown that in thyroid cancer reg γ binds directly to the TGF-β signaling antagonist *SMAD7* and promotes its degradation, leading to the activation of the TGF-β signaling pathway [38]. Furthermore, the SUMO proteins are small protein modifiers that regulate cellular localization and target protein function, playing important regulatory roles in multiple cellular processes, including gene transcription, DNA repair, cell cycle regulation, and apoptosis [39]. Members of the *ZCCHC* gene family were identified by searching the NCBI database with their structural similarity analyzed by MOTIF. The differential expressions of 12 *ZCCHC* gene family members were verified in THCA, confirming their correlation with the main pathogenic genes of THCA. The close association of the *ZCCHC* gene family with THCA was further investigated. The results showed that the *ZCCHC* gene family was closely associated with survival and staging of THCA with its potential as a diagnostic marker for THCA confirmed by the ROC curve.

The correlation analysis showed that the *ZCCHC* gene family was associated with immune function in THCA, which was consistent with the results of previous studies, showing that *ZCCHCs* were associated with immunity. For example, *ZCCHC17* is identified as a novel marker for the diagnosis and prognostic assessment of hepatocellular carcinoma (HCC) by regulating the immune cells in the tumor microenvironment (TME) of HCC patients, with specific reference value for immunotherapy of HCC, suggesting that *ZCCHCs* are associated with immunity [32, 40]. Both GO and PPI network analyses suggested that the *ZCCHC* gene family probably functioned by regulating other proteins through metabolic pathways such as zinc ion binding and transition metal ion binding.

## Conclusion

In conclusion, we have investigated the relationship between the *ZCCHC* gene family and THCA immune function, and identified 12 *ZCCHC* factors as potential diagnostic markers. This could provide new insights for the diagnosis and treatment of THCA. However, there still are some limitations in our study. The molecular data downloaded from the TCGA database contained only a few thyroid adenoma genes, and the *ZCCHC* genes do not regulate all tumor-associated immune cells. In addition, unfortunately, our laboratory was closed due to the COVID pandemic, preventing further experiments which are necessary to validate the findings revealed and the hypothesis proposed in our study.

### Electronic supplementary material

Below is the link to the electronic supplementary material.

**Supplementary Material 1: Table S1.** Primers and their sequences used in the quantitative real-time PCR of ZCCHC gene families

**Supplementary Material 2: Table S2.** Differential genes and functional enrichment analysis

**Supplementary Material 3: Table S3.** ZCCHC12 expression and its correlation with THCA survival and staging

**Supplementary Material 4: Table S4.** PPI network of ZZCH12 and its correlation with immune and tumor stemness and tumor mutational load

**Supplementary Material 5: Table S5.** Chromosomal location and MOTIF structure of the ZCCHC gene family and its correlation and expression with THCA

**Supplementary Material 6: Table S6.** Association of the ZCCHC gene family with THCA survival and staging

**Supplementary Material 7: Table S7.** Relevance of the ZCCHC gene family in THCA to immunity

**Supplementary Material 8: Table S8.** Tumor stemness differences in THCA and their correlation with ZCCHC12

**Supplementary Material 9: Table S9.** GO analysis and PPI network of the ZCCHC gene family

**Supplementary Material 10: Table S10.** Raw data of RT-PCR

## Data Availability

All the datasets used and analyzed during the current study are downloaded from the Cancer Genome Atlas database (https://portal.gdc.com/) and GTEx databases (https://www.genome.gov/Funded-Programs-Projects/Genotype-Tissue-Expression-Project) which are available from the corresponding authors on reasonable request.

## Abbreviations

ZCCHC: zinc-finger CCHC-type
HGNC: HUGO Gene Nomenclature Committee
THCA: thyroid carcinoma
OS: overall survival
TIIC: tumor-infiltrating immune cells
TMB: tumor mutational burden correlation
DEGs: Differentially Expressed Genes
GO: Gene Ontology
KEGG: Kyoto Encyclopedia of Genes and Genomes
PPI: Protein-protein Interaction
OCLR: logistic regression machine learning algorithm
HCC: hepatocellular carcinoma
TME: tumor microenvironment

## References

[1] Nilsson M, Fagman H (2017). Development of the thyroid gland. Development.

[2] Mitchell A, Gandhi A, Scott-Coombes D, Perros P (2016). Management of thyroid cancer: United Kingdom National Multidisciplinary guidelines. J Laryngol Otol.

[3] Rahbari R, Zhang L, Kebebew E (2010). Thyroid cancer gender disparity. Future oncology. (London England).

[4] Cao L, Peng X, Xie J, Yang F, Wen H, Li S (2017). The relationship between iodine intake and the risk of thyroid cancer: a meta-analysis. Medicine.

[5] Albi E, Cataldi S, Lazzarini A, Codini M, Beccari T, Ambesi-Impiombato F, et al. Radiation and thyroid Cancer. Int J Mol Sci. 2017;18(5). 10.3390/ijms18050911.Epub:28445397.PMID:28445397. 10.3390/ijms18050911 PMC5454824 28445397

[6] Myung S, Lee C, Lee J, Kim J, Kim H (2017). Risk factors for thyroid Cancer: a hospital-based case-control study in Korean adults. Cancer Res Treat.

[7] Nixon I, Suárez C, Simo R, Sanabria A, Angelos P, Rinaldo A (2016). The impact of family history on non-medullary thyroid cancer. Eur J Surg Oncology: J Eur Soc Surg Oncol Br Association Surg Oncol.

[8] Araque K, Gubbi S, Klubo-Gwiezdzinska J (2020). Updates on the management of thyroid Cancer. Hormone and Metabolic research=Hormon- und Stoffwechselforschung=Hormones et metabolisme.

[9] Callender G, Carling T, Christison-Lagay E, Udelsman R (2014). Surgery for thyroid cancer. Endocrinol Metab Clin North Am.

[10] Biondi B, Cooper D (2019). Thyroid hormone suppression therapy. Endocrinol Metab Clin North Am.

[11] Lamonica D (2004). Iodine 131 ((131)I) as adjuvant therapy of differentiated thyroid cancer. Surg Oncol Clin N Am.

[12] Powell C, Newbold K, Harrington K, Bhide S, Nutting C (2010). External beam radiotherapy for differentiated thyroid cancer. Clin Oncol (R Coll Radiol (G B)).

[13] Cabanillas M, Ryder M, Jimenez C (2019). Targeted therapy for advanced thyroid Cancer: kinase inhibitors and Beyond. Endocr Rev.

[14] Gao Y, Liu P, Shi R (2020). Anlotinib as a molecular targeted therapy for tumors. Oncol Lett.

[15] Yoon J, Lee E, Koo J, Yoon J, Nam K, Lee J (2020). Artificial intelligence to predict the BRAFV600E mutation in patients with thyroid cancer. PLoS ONE.

[16] Salvatore D, Santoro M, Schlumberger M (2021). The importance of the RET gene in thyroid cancer and therapeutic implications. Nat Reviews Endocrinol.

[17] Rossi M, Buratto M, Tagliati F, Rossi R, Lupo S, Trasforini G (2015). Relevance of BRAF(V600E) mutation testing versus RAS point mutations and RET/PTC rearrangements evaluation in the diagnosis of thyroid cancer. Thyroid: Official Journal of the American Thyroid Association.

[18] Naoum G, Morkos M, Kim B, Arafat W (2018). Novel targeted therapies and immunotherapy for advanced thyroid cancers. Mol Cancer.

[19] Moretti S, Menicali E, Nucci N, Guzzetti M, Morelli S, Puxeddu E (2020). THERAPY OF ENDOCRINE DISEASE Immunotherapy of advanced thyroid cancer: from bench to bedside. Eur J Endocrinol.

[20] Aceituno-Valenzuela U, Micol-Ponce R, Ponce M (2020). Genome-wide analysis of CCHC-type zinc finger (ZCCHC) proteins in yeast, Arabidopsis, and humans. Cell Mol Life Sci.

[21] Tweedie S, Braschi B, Gray K, Jones T, Seal R, Yates B (2021). Genenames.org: the HGNC and VGNC resources in 2021. Nucleic Acids Res.

[22] Lian H, Wei J, Zang R, Ye W, Yang Q, Zhang X (2018). ZCCHC3 is a co-sensor of cGAS for dsDNA recognition in innate immune response. Nat Commun.

[23] Gaza A, Fritz V, Malek L, Wormser L, Treiber N, Danner J (2021). Identification of novel targets of miR-622 in hepatocellular carcinoma reveals common regulation of cooperating genes and outlines the oncogenic role of zinc finger CCHC-type containing 11. Neoplasia (New York NY).

[24] Shi X, Jiang B, Liu H, Fan C (2019). ZCCHC9 promotes proliferation and invasion of Lung cancer through regulating the JNK pathway. J Cell Biochem.

[25] Li T, Fu J, Zeng Z, Cohen D, Li J, Chen Q, et al. TIMER2.0 for analysis of tumor-infiltrating immune cells. Nucleic Acids Res. 2020;48. 10.1093/nar/gkaa407.Epub:32442275.PMID:32442275. W509-W14. 10.1093/nar/gkaa407 PMC7319575 32442275

[26] Chen C, Chen H, Zhang Y, Thomas H, Frank M, He Y (2020). TBtools: an integrative Toolkit developed for interactive analyses of big Biological Data. Mol Plant.

[27] Laha D, Nilubol N, Boufraqech M (2020). New therapies for advanced thyroid Cancer. Front Endocrinol.

[28] Zhu H, Chen K, Chen Y, Liu J, Zhang X, Zhou Y (2022). RNA-binding protein ZCCHC4 promotes human cancer chemoresistance by disrupting DNA-damage-induced apoptosis. Signal Transduct Target Therapy.

[29] Cui Y, Dong Y (2022). ZCCHC12 promotes the progression of osteosarcoma via PI3K/AKT pathway. Aging.

[30] Li Z, Li Z, Wang L, Long C, Zheng Z, Zhuang X (2019). ZCCHC13-mediated induction of human Liver cancer is associated with the modulation of DNA methylation and the AKT/ERK signaling pathway. J Translational Med.

[31] Shi X, Han X, Cao Y, Li C, Cao Y (2021). ZCCHC14 regulates proliferation and invasion of non-small cell Lung cancer through the MAPK-P38 signalling pathway. J Cell Mol Med.

[32] Liu F, Liang J, Long P, Zhu L, Hou W, Wu X (2021). ZCCHC17 served as a predictive biomarker for prognosis and immunotherapy in Hepatocellular Carcinoma. Front Oncol.

[33] Thorsson V, Gibbs D, Brown S, Wolf D, Bortone D, Ou Yang T (2018). The Immune Landscape of Cancer. Immunity.

[34] Ott P, Bang Y, Piha-Paul S, Razak A, Bennouna J, Soria J (2019). T-Cell-inflamed gene-expression Profile, programmed death Ligand 1 expression, and Tumor Mutational Burden Predict Efficacy in patients treated with Pembrolizumab Across 20 cancers: KEYNOTE-028. J Clin Oncology: Official J Am Soc Clin Oncol.

[35] Wang O, Zheng Z, Wang Q, Jin Y, Jin W, Wang Y (2017). ZCCHC12, a novel oncogene in papillary thyroid cancer. J Cancer Res Clin Oncol.

[36] Andriescu E, Căruntu I, Giuşcă S, Lozneanu L, Ciobanu Apostol D (2018). Prognostic significance of cell-adhesion molecules in histological variants of papillary thyroid carcinoma. Romanian J Morphology Embryology = Revue Roumaine de Morphologie et embryologie.

[37] Hou X, Chen C, Lan X, He X (2022). SIGLEC15Unveiling the molecular features, relevant immune and clinical characteristics of in thyroid cancer. Front Immunol.

[38] Jiao C, Li L, Zhang P, Zhang L, Li K, Fang R (2020). REGγ ablation impedes dedifferentiation of anaplastic thyroid carcinoma and accentuates radio-therapeutic response by regulating the Smad7-TGF-β pathway. Cell Death Differ.

[39] Tuccilli C, Baldini E, Sorrenti S, Di Gioia C, Bosco D, Ascoli V, PAPILLARY THYROID CANCER IS CHARACTERIZED BY ALTERED EXPRESSION OF GENES INVOLVED IN THE SUMOYLATION PROCESS (2015). J Biol Regul Homeost Agents.

[40] Leiter A, Gnjatic S, Fowkes M, Kim-Schulze S, Laface I, Galsky M (2020). A COMMON PITUITARY AUTOANTIBODY IN TWO PATIENTS WITH IMMUNE CHECKPOINT INHIBITOR-MEDIATED HYPOPHYSITIS: ZCCHC8. AACE Clin case Rep.

